# Genetic and Environmental Predictors of Adolescent PTSD Symptom Trajectories Following a Natural Disaster

**DOI:** 10.3390/brainsci9060146

**Published:** 2019-06-20

**Authors:** Christina M. Sheerin, Laurel V. Kovalchick, Cassie Overstreet, Lance M. Rappaport, Vernell Williamson, Vladimir Vladimirov, Kenneth J. Ruggiero, Ananda B. Amstadter

**Affiliations:** 1 Virginia Institute for Psychiatric and Behavioral Genetics, Virginia Commonwealth University, Richmond, VA 23298, USA; kovalchicklv@mymail.vcu.edu (L.V.K.); overstreetcm@mymail.vcu.edu (C.O.); Lance.Rappaport@uwindsor.ca (L.M.R.); vladimir.vladimirov@vcuhealth.org (V.V.); Ananda.Amstadter@vcuhealth.org (A.B.A.); 2 Department of Psychology, University of Windsor, Windsor, ON N9B 3P4, Canada; 3 Molecular Diagnostics Laboratory, Virginia Commonwealth University, Richmond, VA 23298, USA; Vernell.Williamson@vcuhealth.org; 4 Departments of Nursing and Psychiatry, Medical University of South Carolina, Charleston, SC 29425, USA; ruggierk@musc.edu

**Keywords:** PTSD, longitudinal, natural disaster trauma, G × E, rare variants, adolescent risk

## Abstract

Genes, environmental factors, and their interplay affect posttrauma symptoms. Although environmental predictors of the longitudinal course of posttraumatic stress disorder (PTSD) symptoms are documented, there remains a need to incorporate genetic risk into these models, especially in youth who are underrepresented in genetic studies. In an epidemiologic sample tornado-exposed adolescents (*n* = 707, 51% female, *M*_age_ = 14.54 years), trajectories of PTSD symptoms were examined at baseline and at 4-months and 12-months following baseline. This study aimed to determine if rare genetic variation in genes previously found in the sample to be related to PTSD diagnosis at baseline (*MPHOSPH9*, *LGALS13*, *SLC2A2*), environmental factors (disaster severity, social support), or their interplay were associated with symptom trajectories. A series of mixed effects models were conducted. Symptoms decreased over the three time points. Elevated tornado severity was associated with elevated baseline symptoms. Elevated recreational support was associated with lower baseline symptoms and attenuated improvement over time. Greater *LGLAS13* variants attenuated symptom improvement over time. An interaction between *MPHOSPH9* variants and tornado severity was associated with elevated baseline symptoms, but not change over time. Findings suggest the importance of rare genetic variation and environmental factors on the longitudinal course of PTSD symptoms following natural disaster trauma exposure.

## 1. Introduction

Approximately 70% of the general population endorse experiencing a traumatic event in their lifetime, with 7% experiencing a natural disaster (e.g., tornado, floods; [1]). Exposure to natural disasters, like other traumas, can lead to a variety of negative mental health outcomes, including posttraumatic stress disorder (PTSD). The prevalence of PTSD following disaster has been estimated at up to 40% among victims, 10–20% among rescue workers, and 5–10% in the general population [2,3]. It has generally been found that, relative to the risk of adult survivors, youth show greater risk of severe impairment following disasters (see review by [4]). Numerous studies of childhood PTSD following natural disasters have estimated prevalence to be over 30% [5,6,7] and demonstrated that these events can have a long-lasting impact (e.g., more than a year following a disaster; [8]). Much of the extant work, however, is cross-sectional and there remains a need to examine the course of symptoms over time following a natural disaster.

Longitudinal studies are important for understanding PTSD risk due to both the chronic nature of the disorder (e.g., [4,9]) and evidence of natural improvement in symptoms without treatment over time [10]. These results are also supported by mixture modeling studies finding that symptom severity can be mapped over time to form distinct trajectory groups (e.g., late-onset and low-stable symptoms; [11]). Beyond better understanding of the longitudinal course of PTSD symptoms following trauma, identifying risk and protective factors associated with these symptom trajectories may lead to improved treatment and prevention strategies through more precise targeting of at-risk groups.

There is a well-established literature on the factors associated with risk for PTSD, and two factors consistently associated with PTSD are trauma severity and social support (or, lack thereof). In a meta-analysis of risk factors for PTSD, factors relating to events during and after the trauma, specifically greater trauma severity, lack of social support, and more subsequent life stress, were associated with the strongest risk for PTSD [12,13]. The association between low levels of social support and greater PTSD risk has also been demonstrated in adolescents exposed to a natural disaster specifically [14]. Alternatively, research has shown that high levels of social support may help individuals cope with traumatic experiences [15]. 

In addition to environmental risk factors, evidence from twin (e.g., [16,17]) and molecular studies [18] suggest moderate heritability for PTSD. The molecular genetics literature has examined specific genetic variation in relation to PTSD from candidate gene designs (see review by [19]) and genome-wide association studies (GWAS), including a recent meta-analysis of GWAS samples [18]. While GWAS arrays capture common genetic variation, there is a growing interest in studying rare variants due to the potential for larger effect sizes on outcomes compared to common variants [20]. Exome arrays targeting low frequency and rare variants [21,22] have begun to identify rare variant contributions to schizophrenia [23,24] and addiction [25]. Our group has conducted the first agnostic, exome-wide analyses for PTSD and identified risk variants in genes significantly associated with PTSD diagnosis (*MPHOSPH9*, *LGALS13*, and *SLC2A2*; [26]). The present study represents a follow-up of these findings to further examine these genes, nominated from the agnostic, exome-wide approach, in a longitudinal framework. 

Given the necessary, but not sufficient, role of trauma exposure in the etiology of PTSD, the examination of how genetic risk may interact with various environmental factors to predict PTSD is a fitting approach [27]. As such, gene-by-environment (G × E) designs have been of interest in the PTSD literature (e.g., *5-HTTLPR* and level of trauma exposure [28]; *apoE* gene and number of trauma exposures; [29,30]; social support, trauma exposure, and various genetic risk variants; [31,32]). While some G × E effects have been further supported through meta-analytic approaches (i.e., FKBP5 and childhood trauma; [33]), there are numerous, noted challenges of candidate G × E studies. In particular, the generally inconsistent findings that characterize the candidate molecular G × E literature have resulted in much skepticism, leading to questions that evidence supporting G × E effects may instead reflect publication bias, low statistical power, and a high false discovery rate [34]. Additional limitations that need to be considered in G × E studies include the potential that the use of logistic regression in G × E psychopathology studies may lead to a higher rate of Type I error and the use of dichotomous “case/control” variables can lead to spurious interaction effects [35], highlighting the potential benefits of examining continuous symptom count, as done in the present study. 

The present study design overcomes some of the G × E limitations in that although it is not a particularly large sample for genetic studies, it is large in comparison to many G × E studies, the genes examined were determined from an agnostic assessment, and symptom count (both level and slope) is the outcome of interest. Moreover, few studies have specifically looked at genetic influences in adolescents, leaving gaps in our understanding of these influences, and the interaction with environmental factors, over time. We sought to extend the current literature by examining the association of well-supported environmental variables on PTSD symptom count over time and determining whether the addition of rare genetic variants nominated by an agnostic approach, alone and in combination with environmental variables, adds to the prediction. Variation in three genes examined in the present study has been shown to be related to PTSD diagnosis at baseline ([26] see Table A1); this study seeks to extend this work by examining longitudinal effects of these genes, and their interplay with environmental factors, on PTSD symptoms. It was hypothesized that the environmental variables and genetic risk scores would be associated with both baseline and trajectory of symptom count. It was further hypothesized that genetic risk would interact with tornado severity to increase PTSD symptoms and that social support would buffer the effects of genetic risk on symptoms.

## 2. Methods

### 2.1. Sample and Procedure

All procedures and protocols for this study were approved by the Institutional Review Board (IRB) at the Medical University of South Carolina. Verbal informed consent/assent was obtained from parents and adolescents prior to participating in the study. Two thousand unrelated families from households affected by the 2011 tornados in Joplin, Missouri and northern Alabama were contacted through phone call or letter. Detailed information on study recruitment and procedures is presented elsewhere [36]. Briefly, caregivers were eligible for the study if they were the guardian of an adolescent aged 12–17 years, had internet access in their home and had access to a telephone. One caregiver and one randomly chosen adolescent in each family was interviewed via phone. Baseline telephone interviews occurred between September 2011 and June 2012, between 4–13 months following tornado exposure. Additional follow-up phone assessments were conducted at 4-months and 12-months post-baseline interview, with the final follow-up interview completed in August of 2013. 

The baseline phone assessment collected information on demographic and environmental variables. PTSD symptoms were assessed during the baseline interview and reassessed during the 4- and 12-month follow-up interviews. Following the completion of the baseline interview, participants were mailed a package that included reimbursement for the interview, a letter informing them about the optional genetic component of the study, a saliva collection kit with detailed instructions for use, and a self-addressed postage paid envelope for mailing the saliva specimen to the laboratory. 

As the overarching study was also designed to examine the efficacy of a self-help web-based resource in the prevention of post-disaster mental health problems, participants were also invited to access the Bounce Back Now website, a brief, interactive web-based intervention (described in detail in [36]). Each participant was randomly assigned to one of three different web intervention groups: 1) web-based invention for adolescents only, 2) web-based intervention for both adolescents and parents, and 3) assessment-only comparison. The two active intervention groups included five evidence-informed modules that provided education and recommendations on strategies for addressing post-disaster mental health areas. Full intervention details and overall outcomes have been previously reported [37]; this work identified a reduction in PTSD symptoms in those who received the web-based intervention at 12-month follow-up. Thus, this was included in all models as a covariate (with both adolescent only and adolescent and parent web-based intervention groups combined, *n* = 480, assessment only *n* = 227, in the current study subsample). A recruitment and assessment flowchart for the overarching study has been published (http://clinicaltrials.gov; NCT01606514) and Figure S1 presents additional information for the present study. 

### 2.2. Participants

Adolescents between the ages of 12–17 with tornado exposure and who provided genotypic data that passed quality control standards (reported below, Section 2.3.1.) were included in the current study. Of the 2000 participants who completed the parent study, 780 returned a DNA sample of which 763 were successfully genotyped. Of the genotyped samples, 707 passed quality control checks and were utilized in the present study. There were no differences between those who returned samples for genotyping and those who did not on age or sex. However, those who did return saliva samples were more likely (all *p* values < 0.05) to meet criteria for PTSD, endorsed greater PTSD symptoms, and to be African-American, compared to those who did not return saliva samples. Further, they also endorsed lower emotional support and recreational support items. However, all of these significant differences between groups were small effects (all *d* values < 0.15). 

### 2.3. Genetic Data Collection and Analysis

#### 2.3.1. DNA Collection, Genotyping, and Quality Control

Oragene kits (DNA Genotek, Ottawa, ON, Canada) were used for collection of saliva and gene extraction and isolation was conducted at Yale University. The average yield is generally 110 μg of DNA from each sample, and the failure rate is about 3%. Genotyping was then completed via the Illumina Human Exome BeadChip (Illumina, Inc., San Diego, CA, USA), which queries 247,870 variable exonic sites, using standard protocols suggested by the manufacturer. Variants (single nucleotide polymorphisms; SNPs) with call rates (< 95%) and deviations from Hardy-Weinberg equilibrium (*p* < 10^−6^) were eliminated, resulting in a final sample of 707 that passed all quality control procedures and used for all genetic analyses. 

#### 2.3.2. Ancestry Determination 

Self-reported ancestry was verified using a randomly selected subset of SNPs, and a Principal Components Analysis (PCA) in the larger sample was conducted on 9827 SNPs known to differentiate members of different population groups. The PCA yielded four components that captured greater than 96% of the variability. The two principle components with eigenvalues of 1 or higher were retained. The first two PCs explained 88% of the variance in self-reported ethnicity and were used in genetic analyses to control for population stratification. 

#### 2.3.3. Creation of Gene-based Risk Scores of Rare Variants

As is common in rare-variant analyses, to increase power, gene-based tests were performed (i.e., using summed risk scores based on the presence of minor alleles in rare variants across a gene; [38]). Previous work by our group ([26]; details available in Supplemental Materials Method) conducted rare-variant association tests of gene-based models to investigate for rare variant effects on PTSD diagnostic status, adjusting for ancestral PCs, age, sex, and previous trauma history. Analyses were conducted using the sequence kernel association test (SKAT; [39]). It is noted that African (AA) and European (EA) ancestry groups were combined to increase power given sample size; analyses [26] determined that there were no group differences in allele frequency. This work identified variants in three genes associated with PTSD diagnosis in this sample that survived the multiple testing correction: *MPHOSPH9*, *LGALS13S13*, and *SLC2A2*. The summed score of rare variants in each of these three genes were used in subsequent study analyses examining association with PTSD symptom count over time, described below (Section 3.3.). For *MPHOSPH9*, sum scores ranged from 0 to 2 risk variants (with 13% of the sample having at least one variant), for *LGALS13,* participants had either 0 or 1 (3% of sample) rare variants, and for *SLC2A2*, sum scores ranged from 0–4 risk variants (38% had at least one variant). 

## 3. Measures 

### 3.1. Outcome Variable

#### Adolescent Symptoms of PTSD

The PTSD module from the National Survey on Adolescents [40] assessed for DSM-IV PTSD symptoms and diagnostic status. For present analyses, the total sum of 17 PTSD symptoms endorsed in the past 4 weeks at each time point (baseline/post-tornado, 4-month, and 12-month follow-up interviews) was used. The mean symptom count at baseline in the sample was 3.69 (SD = 4.02) and 16.6% of participants met diagnostic criteria for PTSD (mean in PTSD cases was 10.59, SD = 2.69). Chronbach’s alphas at each time point were 0.80, 0.88, 0.89, respectively. 

### 3.2. Environmental Variables

#### 3.2.1. Tornado Severity

A tornado severity assessment was created for this study, consisting of parental report on nine yes/no items, as follows: whether or not the adolescent was physically injured by the tornado; was concerned about the safety of others; and experienced damage to the following: their home, furniture, sentimental objects, vehicles, pets, land; and any other item not mentioned. A sum score of endorsed items was used in the present study. While total tornado severity could range from 0 to 10 consequences, participants in the present sample endorsed severity ranging from 0 to 9, with a mean of 3.32 (SD = 1.94).

#### 3.2.2. Social Support

The Social Support Scale [41] was used to assess social support adolescents received from their mother, father, siblings, friends, and other peers. Items assessed the extent to which adolescents believed others were helpful when they had a personal problem (emotional support) and wanted to have fun (recreational support). Answer choices were “not at all”, “somewhat”, or “a great deal” (scored 0–2). Responses of support received from these five sources were summed to create scores on emotional (mean = 6.9, SD = 1.9, range = 0–10) and recreational (mean = 8.1, SD = 1.7, range = 2–10) supports. Of note, the reliability of the scales in the present study sample was fairly low (*a* = 0.56 recreational support; *a* = 0.52 for emotional support; *a* = 0.71 total) and similar to that in the larger, parent study, which is contrary to existing literature on the psychometric properties of this scale [41], although this work didn’t examine social support types separately. 

### 3.3. Analytic Plan

The present study examined inter-individual (i.e., between-person) correlates of baseline symptom severity and moderators of symptom change over time. A logistic mixed effects (i.e., multilevel) model for PTSD diagnosis demonstrated an overall reduction over time in the likelihood that participants met criteria for a diagnosis of PTSD. However, there was insufficient between-person variability in the trajectory of diagnostic change to examine moderators of change in the diagnosis. Specifically, a non-positive definite variance-covariance matrix indicated little between-person variance in the linear slope over time. We note that binary variables (e.g., diagnoses) show reduced variance as compared to continuous indices of symptom severity. As a continuous variable, symptom severity provides more information, particularly regarding the trajectory of symptom severity over time, which facilitates examining between-person moderators of symptom change over time. 

Analyses proceeded in three steps to examine the influence of environmental variables (i.e., tornado severity, recreational support, emotional support) and rare variant scores in putative genes (i.e., *MPHOSPH9*, *LGALS13*, and *SLC2A2*) on PTSD symptom change over time. Initially, a mixed effects (i.e., multilevel) model examined overall linear and quadratic change in PTSD symptom severity over time. Subsequently, the inclusion of a random intercept and linear slope permitted examining between-person variability in baseline PTSD symptom severity and in change over time, respectively. 

Analyses began with a base model that included a random intercept, random linear slope, and covariates (i.e., age, sex, web-based intervention group). Next, environmental factors (i.e., recreational support, emotional support, tornado severity) were incorporated into the model. Three separate models were then created for each index of genetic risk. Each model included covariates and environmental factors to examine whether genetic load influenced baseline symptom severity, PTSD symptom change over time, and the potential interaction of each environmental factor with genetic load to predict baseline severity and symptom change over time. Analyses were run with SAS software version 9.4 (SAS Institute Inc., Cary, NC, USA, 2013) using the MIXED procedure with maximum likelihood estimation and Kenward-Rodger degrees of freedom. Logistic mixed effects regression analyses used the GLIMMIX procedure.

## 4. Results 

Descriptive statistics and correlations among the demographic and environmental variables of interest are presented in Table 1. 

### 4.1. Unconditional Model and Individual Differences in Baseline Severity 

Prior to the analyses described above, an unconditional model with no independent variables was fit to estimate the proportion of the total variance in symptom severity explained at each level of analysis (e.g., within-person change over time). 

Results indicated that the total variance in PTSD symptom severity was attributable to both between-person (52.80%) and within-person (47.20%) variation. Linear, quadratic, and cubic effects for time were added sequentially to determine a best-fitting model to describe change over time. Overall, as demonstrated by the linear slope, PTSD symptoms severity decreased monotonically downward over time (*B* = −0.50, 95% CI = −0.61, −0.39. *p* < 0.001; see Model 1 in Table 2). The statistically significant quadratic slope indicates further deceleration of symptom change over time; participants show greater decrease in PTSD symptoms from baseline to the 4-month assessment period than between the 4- and 12-month assessment periods. Among covariates, older age was associated with elevated baseline symptom severity (*B* = 0.17 95%, *p* < 0.05).

### 4.2. Environmental Factors Model

When incorporated into the model (see Model 2 in Table 2), lower recreational support (*B* = −0.50, 95% CI = −0.71, −0.30, *p* < 0.001) and greater tornado severity at baseline (*B* = 0.29, 95% CI = 0.14, 0.43, *p* < 0.001) were associated with elevated baseline PTSD symptom severity. Recreational support at baseline also predicted attenuated symptom change over time (*B* = 0.03, 95% CI = 0.01, 0.05, *p* < 0.05).

### 4.3. Gene Models

*MPHOSPH9* was not significantly associated with baseline PTSD symptoms or symptom change over time (see Table 3). However, *MPHOSPH9* moderated the association of tornado severity with PTSD symptoms at baseline: participants with more variants within the *MPHOSPH9* gene showed a stronger association of tornado severity with baseline PTSD symptoms (*B* = 0.49, 95% CI = 0.10, 0.87, *p* < 0.05). Three-way interactions among linear slope, *MPHOSPH9* variants, and each of the environmental factors were not significant, which indicates little evidence of an interaction of *MPHOSPH9* variants with environmental factors to moderate symptom change over time (see Table 3 and Table A1 for detailed results). 

*LGALS13* was not significantly associated with baseline PTSD symptoms; however, elevated *LGALS13* variants predicted attenuated PTSD symptom change over time (*B* = 0.17, CI = 0.01, 0.32, *p* < 0.05; see Table 3). No other significant interactions or three-way interactions among linear slope, *LGALS13* variants, and each of the environmental factors were identified.

*SLC2A2* was not significantly associated with baseline PTSD symptoms or PTSD symptom change over time (see Table 3). Moreover, no significant interactions were identified.

## 5. Discussion

The present study sought to expand upon the existing literature and previous gene-based findings by our group to determine the longitudinal association of environmental variables and impact of rare variant genetic risk, and their interaction, on PTSD symptoms over time in an adolescent, tornado-exposed sample (*N* = 707). The environmental variables examined are well-established, and the rare variant gene-based risk scores examined were nominated from an agnostic approach. Overall, symptom count in this sample was low, as would be expected in a population-based study, with rates of PTSD diagnosis at 16.6%, in line with other disaster-exposed samples [42]. However, findings indicated that symptoms differed significantly both within and across participants and there was a significant decrease in symptom count over time (across the three assessments, baseline, 4 months, 12 months), controlling for intervention effects. Of the demographic variables examined, results suggested that while older age was associated with greater initial symptoms, it was also associated with greater decrease in symptoms over time, suggesting older adolescents may be more likely to process appropriately or utilize protective factors in a more effective manner. While a ‘basement effect’ cannot be ruled out for younger age, analytic approaches used in the present study help account for this possibility, suggesting age is relevant for symptom change, and not a statistical artifact.

At baseline, consistent with hypotheses, tornado severity was associated with increased PTSD symptom count, and recreational support was associated with decreased symptom count. Findings broadly align with extant literature suggesting the relevance of trauma severity on negative outcomes and social support as a buffer against PTSD (e.g., [15]). The finding of an association of recreational support but not emotional support suggests specific benefits for positive activity. Indeed, a small body of literature exists examining the potential benefits of behavioral activation (a behaviorally based treatment approach) in assisting with distress following loss by helping individuals engage in their social environment and increase contact with positive reinforcement [43,44]. 

Although associated with PTSD at baseline, tornado severity did not have a lasting effect on outcomes, as it was not associated with change in symptom count over time. Recreational support was also significantly associated with symptom change over time; however, contrary to expectations, it was associated with attenuated symptom change (i.e., less improvement) over time. It may be that while recreational support is protective in the immediate aftermath of a trauma, it may hinder full recovery, perhaps due to prevention of processing emotions, an increase activities that are not necessarily helpful, or the source of support (i.e., family versus peers). Further research on elements of recreational support that are helpful and/or potentially harmful following trauma exposure is warranted.

Although genetic risk variants may have been associated with PTSD diagnosis at baseline per previous work, they were not associated with overall symptom count at baseline. This may be due to the low symptom count in the sample as a whole or reflect that genetic association with clinical diagnosis cannot necessarily be equated with the presence of symptoms of the disorder. However, *LGALS13* was associated with attenuated PTSD symptom change over time; individuals with the risk variant in this gene evidenced less symptom decrease over time. The *LGALS13* gene has been shown to regulate innate and adaptive immune responses [45], a finding that aligns with existing GWAS findings of common variants and expression studies finding association with PTSD in genes and pathways related to the immune response [46,47,48]. Findings suggest that variation in this gene may be relevant for how individuals respond over time, and may represent a particularly at-risk group for longer-term negative outcomes following trauma.

Contrary to hypotheses, there was limited evidence of an interaction between any of the genetic risk variables and environmental variables on symptom count at baseline or over time, with the exception of a significant interaction between *MPHOSPH9* and tornado symptom severity at baseline. Individuals with more rare variants (i.e., greater genetic risk) in this gene and greater tornado severity evidenced higher symptom severity at baseline. While there is a large literature linking *MPHOSPH9* to health conditions such as type 2 diabetes [49] and multiple sclerosis [50], this gene has not previously been linked to psychiatric conditions and may warrant further investigation. 

The present study is considered preliminary given sample size limitations, but it is noted that post-disaster genetically-informed studies of PTSD such as this one are useful in that they provide a sample with more consistent time-since-trauma, less likely gene-environment correlation, and trauma exposure can be confirmed in controls. As the extent of exposure can be quantified, it is possible to examine if the degree of exposure moderates genetic effects. Although rare variation in specific genes was examined in this study, these genes were nominated from an agnostic approach in our data. The association of rare variant genetic risk in the gene *MPHOSPH9* with tornado severity adds to the growing body of literature examining how traumatic experiences interact with genetic predisposition to modify risk for complex traits. The examination of molecular genetic risk in combination with environmental factors represents an important extension on existing evidence in the twin literature [51] and continued work is needed to elucidate the potential mechanisms of the trauma exposure and PTSD risk association, if replicated. 

Another strength of the present study is the longitudinal nature of assessment. Expansion of GxE designs to examine trajectories and long-term outcomes will further inform our understanding of this interaction to the general course and trajectory of symptoms. We found evidence of the importance of age, recreational social support, rare variation in *MPHOSPH9* interacting with trauma severity, and rare variation in *LGALS13* on PTSD symptom change over time. Expanding the investigation of important post-trauma environmental factors to those beyond the frequently studied factors of trauma severity and social support is also needed (e.g., parenting style and relationship conflict, particularly relevant in an adolescent population).

A number of limitations to the present work also exist. First, the sample size, while large for examination of phenotypic outcomes, is small for examination of genetic risk and interaction effects, and thus, power is limited. Limited power may particularly impact investigation of 2- and 3-way interactions, with increased likelihood of Type II error. As such, findings should be considered preliminary in nature. Second, while the small number of genes examined were chosen based on an agnostic, empirical approach, this was within the same sample as the current analyses; replication in independent samples is needed. Third, although this work expands the growing body of research on rare variant association with complex psychiatric phenotypes, by nature of rare variation, the range of variation in the studied genes was small. Fourth, while it was noted that AA and EA ancestries were combined, analyses suggested that these groups did not differ in minor allele frequency for these rare variants. Fifth, the significant genetic effects identified in this study are indicative of very small effects, much smaller than psychosocial variables, and given the reasonably lower power, the possibility of false positives is relevant and should be noted. Finally, the examination in adolescents and following a specific trauma adds to methodological rigor, but also limits generalizability. Thus, future research is needed to determine if findings would replicate in adults and following different types of trauma exposure. 

## 6. Conclusions

The current study sought to advance previous literature by exploring PTSD symptom outcomes in a longitudinal manner, examining the association of rare genetic variants, important environmental factors, and their interaction. These preliminary findings suggest the relevance of rare genetic variants, environmental factors, and their interaction, on variation in PTSD symptoms over a 12-month period. Further research in larger samples, and efforts to replicate these findings will add to our understanding of PTSD etiology and course. Continued efforts to identify both common and rare genetic variation associated with the course and trajectory of PTSD outcomes, and how they are impacted by relevant environmental factors, holds promise for future clinical implications, such as determining individuals who should be targeted in primary and secondary prevention efforts in the months following trauma exposure [52] as well as assist in treatment planning, both at the individual and community level.

## Figures and Tables

**Table 1:** Descriptive statistics and correlations among study variables and covariates at baseline.

	Mean (SD)	1.	2.	3.	4.	5.	6.
1. PTSD symptoms	3.69 (4.02)	--					
2. Recreational support	8.10 (1.70)	−0.29 **	--				
3. Emotional support	6.90 (1.98)	−0.16 **	0.57 **	--			
4. Tornado severity	3.28 (3.00)	0.16 **	−0.03	0.05	--		
5. Age	14.5 (1.73)	0.08 *	−0.09 *	0.08 *	0.03	--	
6. Sex ^a^	48.1%	−0.08 *	0.05	−0.05	−0.02	−0.10 *	--
7. Self-reported race ^a^	67.6%	0.04	−0.07	−0.34 *	0.03	0.02	0.04

*, *p* < 0.05; **, *p* < 0.01; ^a^, The reference group for sex is male; the reference group for self-reported race is European American.

**Table 2:** Models including random slopes (model 1) and environmental factors (model 2) predicting posttraumatic stress disorder symptoms (*N* = 707).

	Model 1	Model 2
**Intercept (Baseline)**		
Intercept	4.00 (0.29) ***	3.80 (0.30) ***
Web-based Intervention Group	−0.10 (0.30)	0.02 (0.30)
Sex	−0.50 (0.28)	−0.55 (0.28)
Age	0.17 (0.08) *	0.18 (0.08) *
Recreational Support	--	−0.50 (0.10) ***
Emotional Support	--	−0.12 (0.08)
Tornado Severity	--	0.29 (0.07) ***
**Change (Linear and Quadratic Slope)**		
Linear slope	−0.50 (0.06) ***	−0.51 (0.05) ***
Quadratic slope	0.03 (0.004) ***	0.03 (0.004) ***
Sex × Linear slope	−0.02 (0.03)	−0.01 (0.03)
Age × Linear slope	−0.01 (0.01)	−0.02 (0.01) *
Web-based Intervention Group × Linear slope	−0.06 (0.03) *	−0.04 (0.03)
Recreational Support × Linear slope	--	0.03 (0.01) *
Emotional Support × Linear slope	--	0.01 (0.01)
Tornado Severity × Linear slope	--	−0.01 (0.01)

*, *p* < 0.05; ***, *p* < 0.05. Entries show parameter estimates with standard errors in parentheses. Web-based intervention group = dummy coded to those who did the web-based intervention compared to those who received assessment only, used as a covariate in all analyses.

**Table 3:** Estimates from models including the *MPHOSPH9*, *LGALS13* and *SLC2A2* variants predicting posttraumatic stress disorder symptoms over time (*N* = 707).

	*MPHOSPH9*	*LGALS13*	*SLC2A2*
**Baseline (Intercept)**			
Gene	0.13 (0.38)	−1.52 (0.83)	0.12 (0.12)
Gene × Recreational Support	0.21 (0.24)	0.09 (0.85)	0.02 (0.08)
Gene × Emotional Support	−0.01 (0.20)	−0.02 (0.68)	−0.06 (0.07)
Gene × Tornado Severity	0.49 (0.20) *	0.13 (0.44) ***	0.03 (0.06)
**Change (Linear and Quadratic Slope)**			
Gene × Linear Slope	0.04 (0.04)	0.17 (0.08) *	−0.01 (0.01)
Gene × Recreational Support × Linear Slope	0.01 (0.02)	0.10 (0.08)	0.004 (0.01)
Gene × Emotional Support × Linear Slope	0.01 (0.02)	0.04 (0.07)	0.01 (0.01)
Gene × Tornado Severity × Linear Slope	−0.01 (0.02)	−0.03 (0.04)	−0.001 (0.01)

*, *p* < 0.05; ***, *p* < 0.05. Details from the full model (e.g., including intercept, effects of environmental variables) are available in Table A1.

## Supplementary Materials

The following are available online at https://www.mdpi.com/2076-3425/9/6/146/s1. Figure S1: Flow chart depicting the recruitment and assessment of study participants and genetic data collected used in the present analyses. Note: all families were contacted for follow-up interviews, irrespective of their involvement with the intervention or whether or not they returned saliva samples. Ns shown are only those in the current study analyses (those with genotypic data that passed quality control standards for use in analyses); Supplemental Methods describing details of the DNA collection, genotyping, quality control, and analytic methods and results of the gene based analyses of rare variants that identified the genes used in present study analyses. Further details on the main study model are also presented.

## Appendix A

**Table A1:** Estimates from full models including the *MPHOSPH9*, *LGALS13* and *SLC2A2* variants predicting posttraumatic stress disorder symptoms over time (*N* = 707).

	*MPHOSPH9*	*LGALS13*	*SLC2A2*
**Intercept (Baseline)**			
Intercept	3.79 (0.30) ***	3.82 (0.30) ***	3.76 (0.30) ***
Web-based Intervention Group	0.07 (0.31)	−0.004 (0.30)	0.04 (0.31)
Sex	−0.60 (0.28) *	−0.56 (0.28) *	−0.54 (0.28)
Age	0.17 (0.08) *	0.18 (0.08) *	0.18 (0.08)
Recreational Support	−0.50 (0.10) ***	−0.50 (0.10) ***	−0.48 (0.10) ***
Emotional Support	−0.14 (0.08)	−0.12 (0.08)	−0.12 (0.08)
Tornado Severity	0.27 (0.07) ***	0.28 (0.07) ***	0.29 (0.07) ***
Gene	0.13 (0.38)	−1.52 (0.83)	0.12 (0.12)
Gene × Recreational Support	0.21 (0.24)	0.09 (0.85)	0.02 (0.08)
Gene × Emotional Support	−0.01 (0.20)	−0.02 (0.68)	−0.06 (0.03)
Gene × Tornado Severity	0.49 (0.20) *	0.13 (0.44) ***	0.03 (0.06)
**Change (Linear and Quadratic Slope)**			
Linear slope	−0.52 (0.06) ***	−0.52 (0.05) ***	−0.51 (0.06) ***
Quadratic slope	0.04 (0.004) ***	0.03 (0.004) ***	0.03 (0.004) ***
Sex × Linear slope	−0.01 (0.03)	−0.01 (0.03)	−0.01 (0.03)
Age × Linear slope	−0.02 (0.01) *	−0.02 (0.01) *	−0.02 (0.01)
Gene × Linear Slope	0.04 (0.04)	0.17 (0.08) *	−0.01 (0.01)
Web-based Intervention Group × Linear slope	−0.06 (0.03)	−0.04 (0.03)	−0.04 (0.03)
Recreational Support × Linear slope	0.02 (0.01) *	0.03 (0.01) *	0.02 (0.01) *
Emotional Support × Linear slope	0.01 (0.01)	0.01 (0.01)	0.01 (0.01)
Tornado Severity × Linear slope	−0.01 (0.01)	−0.01 (0.01)	−0.01 (0.01)
Gene × Recreational Support × Linear Slope	0.01 (0.02)	0.10 (0.08)	0.004 (0.01)
Gene × Emotional Support × Linear Slope	0.01 (0.02)	0.04 (0.07)	0.01 (0.01)
Gene × Tornado Severity × Linear Slope	−0.01 (0.02)	−0.03 (0.04)	−0.001 (0.01)

*, *p* < 0.05; ***, *p* < 0.05. Entries show parameter estimates with standard errors in parentheses.
