# Supplementary material for: Genetic and Environmental Predictors of Adolescent PTSD Symptom Trajectories Following a Natural Disaster
Source: Brain Sci. 2019 Jun 20;9(6):146. doi: 10.3390/brainsci9060146 (PMC6627286; doi:10.3390/brainsci9060146)

Supplemental Figure 1. Flow chart depicting the recruitment and assessment of study participants and genetic data collected used in the present analyses. Note: all families were contacted for follow-up interviews, irrespective of their involvement with the intervention or whether or not they returned saliva samples. Ns shown are only those in the current study analyses (those with genotypic data that passed quality control standards for use in analyses).

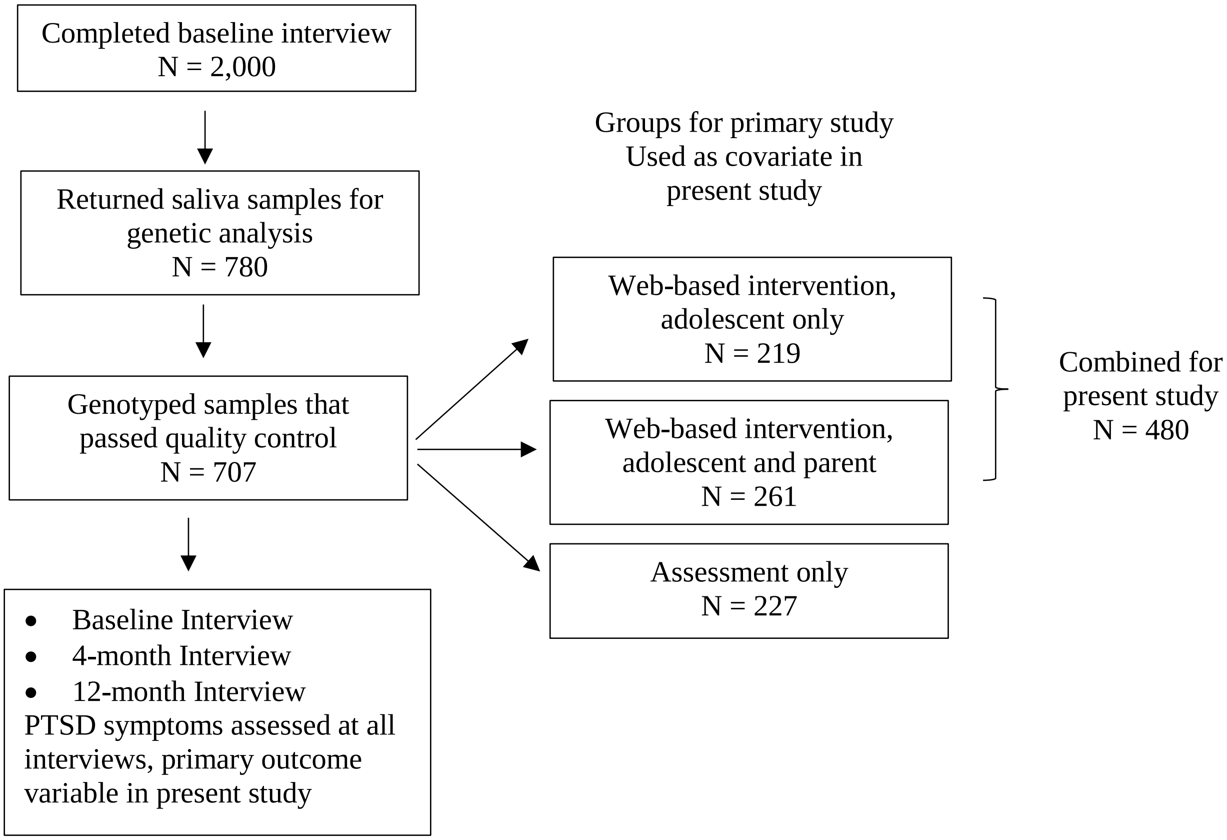

Supplement: Supplementary file 1 [file brainsci-09-00146-s001.zip › brainsci-530529-supple/Supplemental Figure 1.pdf]
