# Supplementary material for: Genetic and Environmental Predictors of Adolescent PTSD Symptom Trajectories Following a Natural Disaster
Source: Brain Sci. 2019 Jun 20;9(6):146. doi: 10.3390/brainsci9060146 (PMC6627286; doi:10.3390/brainsci9060146)
Supplement: Supplementary file 1 [file brainsci-09-00146-s001.zip › brainsci-530529-supple/Supplemental Methods.pdf]

Supplemental Methods

DNA Collection, Genotyping, and Quality Control

Oragene kits were used for collection of saliva, and they were mailed to Yale University for isolation and genotyping. Samples were processed on the Human Exome BeadChip v1.0 (Illumina, INC, San Diego, CA), which queries 247,870 variable exonic site. SNPs with call rates (< 95%) and deviations from Hardy-Weinberg equilibrium ($p < 10^{-6}$) were eliminated. Of the 780 participants who returned samples, 763 were genotyped; of those genotyped, 56 subjects had a GenCall quality score < 0.38 or a call rate < .92 and were excluded from further allele frequency calculations, resulting in 707 samples successfully genotyped by the array.

Self-reported ancestry was verified using a randomly selected subset of SNPs, and a Principal Components Analysis (PCA) in the larger sample (from Ruggiero et al., 2015) was conducted on 9,827 SNPs known to differentiate members of different population groups. The PCA yielded four components that captured greater than 96% of the variability. The two principle components with eigenvalues of 1 or higher were retained. The first two PCs explained 88% of the variance in self-reported ethnicity, respectively, and were used in all analyses to control for population stratification. These two components differentiate European American (EA) and African Americans (AA; current subsample racial composition: 67.3% EA, 29.5% AA, 3.1% Other).

Gene Based Analyses of Rare Variants

All analyses were corrected for multiple testing using the conservative false discovery rate (FDR) Benjamini Hochberg method, with an FDR-corrected significance threshold set at $p_{adj} < .001$. Analyses were adjusted to control for ancestry, age, sex, prior trauma history count, and tornado severity. It is noted that AA and EA ancestry groups were combined to increase power given the sample size. In addition to controlling for ancestry PCs, we also investigated whether there were significant differences in AA and EA allele frequency in the study. Minor allele frequencies (MAF) were extracted from both ancestry groups (based on self-report) and examined with a t-test in R, using mean MAF binned by chromosome; results showed no differences between AA/EA groups. A chi-square test was conducted as a follow-up ($X^2 = 0.0018$, df = 25, p = 1) and also showed no significant group difference.

Rare-variant association tests of gene-based models were conducted for PTSD diagnosis. As is common in rare-variant analyses, to increase power, gene-based tests were performed to investigate the aggregate rare variant effect on PTSD diagnostic status and symptom severity. Gene-based tests have also been shown to accommodate complicated LD structure among SNPs as well as differences in size (Guo, Liu, Wang, & Zhang, 2013) and are believed to be more powerful than a single-variant based test. Analyses for PTSD diagnostic status were conducted using the sequence kernel association test (SKAT; Wu et al., 2011), a variance component test that is quite powerful when the direction of effect is unknown (i.e., for rare variants with both disease-increasing as well as disease decreasing impact).

The relative contributions of rare variants to PTSD diagnosis were assessed with gene boundaries defined as all SNPs that fell within (+/- 20kb) of known gene boundaries. We chose to expand gene boundaries by such a margin as to include any SNPs that might be found within regulatory regions flanking the protein coding sequences, resulting in a mean of 20.58 (SD=8.3) SNPs.

In the analysis with lifetime diagnosis as the phenotype of interest, four genes were significant at the $p_{adj} \leq 0.001$ and many more showed a suggestive trend toward significance (see *Table 1*). The four genes were: M-phase phosphoprotein 9 (*MPHOSPH9*, p_{adj}=.0002), lectin, galactoside binding soluble 13 (*LGALS13*, p_{adj}=.0002), chromosome 12 open reading frame 50 (*C12orf50*, p_{adj}=.0002), and solute carrier family 2, member 2 (*SCL2A2*, p_{adj}=.001). Three of the four genes were carried forward in further analyses; *C12orf50* was not carried forward as there exists no apparent evidence from the literature that seems to implicate it with any psychiatric of physical conditions. Further work is needed to determine if this is a novel finding that can be replicated or an erroneous association. The

remaining three genes represent the predictors in subsequent longitudinal analyses of PTSD symptom count.

Main Study Model

The following equation represents the initial mixed effects (i.e., multilevel) model used to evaluate change in PTSD symptom severity over time (see Raudenbush & Bryk, 2002 for review).
$y_{ij} = \beta_{0j} + \beta_{1j} * Time_j + \beta_{2j} * Time_j^2 + r_{ij}$ where $r_{ij} \sim N(0, \sigma^2)$ and

$$\beta_{0j} = \gamma_{00} + u_{0j}$$

$$\beta_{1j} = \gamma_{10} + u_{1j}$$

where $\begin{bmatrix} u_{0j} \\ u_{1j} \end{bmatrix} \sim N\left(\begin{bmatrix} 0 \\ 0 \end{bmatrix}, \begin{bmatrix} \sigma_{00}^2 & cov_{10} \\ cov_{10} & \sigma_{11}^2 \end{bmatrix}\right)$.

r_{ij} indexes error variance where as u_{0j} and u_{1j} index a random intercept and linear slope, respectively, with a mean of 0, interindividual variance (e.g., σ_{11}^2), and covariance representing the association of baseline PTSD severity with symptom improvement (i.e., cov_{10}; sometimes called 'basement' or 'floor effects'). Subsequent analyses regressed PTSD symptom severity (i.e., y_{ij}), intercept (β_{0j}), and linear slope (β_{1j}) on environmental and genetic factors.

References

Guo, X., Liu, Z., Wang, X., & Zhang, H. (2013). Genetic association test for multiple traits at gene level. *Genet Epidemiol, 37*(1), 122-129. doi:10.1002/gepi.21688

Ruggiero, K. J., Davidson, T. M., McCauley, J., Gros, K. S., Welsh, K., Price, M., . . . Amstadter, A. B. (2015). Bounce Back Now! Protocol of a population-based randomized controlled trial to examine the efficacy of a Web-based intervention with disaster-affected families. *Contemp Clin Trials, 40*, 138-149. doi:10.1016/j.cct.2014.11.018

Raudenbush, S. W., & Bryk, A. S. (2002). *Hierarchical linear models: Applications and data analysis methods* (Vol. 1). Sage.

Wu, M. C., Lee, S., Cai, T., Li, Y., Boehnke, M., & Lin, X. (2011). Rare-variant association testing for sequencing data with the sequence kernel association test. *Am J Hum Genet, 89*(1), 82-93. doi:10.1016/j.ajhg.2011.05.029
